# Supplementary material for: Safety and Immunogenicity of the GamTBvac, the Recombinant Subunit Tuberculosis Vaccine Candidate: A Phase II, Multi-Center, Double-Blind, Randomized, Placebo-Controlled Study
Source: Vaccines (Basel). 2020 Nov 3;8(4):652. doi: 10.3390/vaccines8040652 (PMC7712213; doi:10.3390/vaccines8040652)
Supplement: Supplementary file 1 [file vaccines-08-00652-s001.pdf]

Table S1. Inclusion, non-inclusion, and exclusion criteria.

**Inclusion criteria**	To participate in the study, a volunteer must meet all of the following criteria:<br>1. Ability to understand the research requirements, signed informed consent form and consent to any restrictions applicable during the study.<br>2. The age of volunteers is from 18 to 49 years inclusive at the time of signing the informed consent.<br>3. Volunteers who are completely healthy based on a medical assessment, including medical history, physical examination, and laboratory examination.<br>4. Absence of signs of lung disease, confirmed by chest x-ray.<br>5. Body mass index (BMI) from 18 to 30 kg / m2, inclusive.<br>6. For female volunteers: women are eligible if they are not pregnant (pregnancy test (test strip) is negative at screening and on the day 0), no lactation, and at least one of the following conditions applies: a) no reproductive potential or b) women with preserved reproductive potential who agree to follow contraceptive recommendations throughout the study.<br>7. BCG vaccination was performed, which is documented in the medical history or the presence of a scar.<br>8. Positive result of PPD skin test: induration ≥ 5 mm and ≤ 9 mm.<br>9. The absence of markers of the immune response to the mycobacterial proteins ESAT6 and CFP10 in the Diaskintest and QuantiFERON TB Gold ELISA tests simultaneously at screening. Immune response to that proteins characterize the probable contact with M. tuberculosis prior to the start of the study.<br>10. No fact of living or working with someone diagnosed with TB within 3 months prior to day 0 of the study.<br>11. Absence of malignant neoplasms at the moment and within 5 years prior to inclusion in the study.<br>12. Absence of malignant blood diseases.
**Non-inclusion criteria**	Volunteers who have at least one of the criteria listed below should not be included in the study:<br>1. Presence of symptoms of acute disease, including temperature in the armpit > 37.5 °C, within 5 days before the start of the study and on day 0 of the study.<br>2. History or presence of tuberculosis, including extrapulmonary tuberculosis.<br>3. Negative PPD skin test (<5 mm) or hyperergic positive PPD skin test results (> 9 mm).<br>4. Positive or questionable result in the Diaskintest and / or QuantiFERON TB Gold ELISA tests.<br>5. History or presence of autoimmune diseases or immunosuppression, or family history of congenital or hereditary immunodeficiency.<br>6. Positive test results for HIV-1/2 antibodies, HBsAg or hepatitis C antibodies at screening.<br>7. Use of immunosuppressive or other immunomodulatory medications within 42 days prior to day 0 of the study.<br>8. Use of immunoglobulin or blood products within 180 days before day 0 of the study, or scheduled administration during the study.<br>9. Use of any investigational drug or investigational vaccine within 90 days prior to the day of screening, or planned participation in other clinical trials during the study.<br>10. Use of antibacterial drugs within 14 days before day 0 of the study (oral administration) or within 28 days before day 0 of the study (parenteral administration).

	11. Use of the study GamTBvac vaccine at any time prior to day 0 of the study.<br>12. Planned use/administration of the registered vaccine within 28 days before and 28 days after vaccination with the investigational vaccine.<br>13. Planned surgical intervention (on a scheduled basis) during the study.<br>14. History or laboratory evidence of any possible immunodeficiency condition.<br>15. History of allergic diseases or reactions that may be aggravated by any component of the investigational vaccine.<br>16. History of diseases that could compromise the safety of the study participant, including but not limited to: impairment of lung function in any lung disease, heart or kidney failure, neurological disease, epilepsy or infant seizures, diabetes, cancer.<br>17. History or presence of any systemic disease or any chronic disease that, in the opinion of the investigator, may affect the assessment of the safety or reactogenicity or immunogenicity of the investigational vaccine.<br>18. History of chronic alcohol or drug abuse.<br>19. History or presence of diseases or skin features that, in the opinion of the investigator, may affect the assessment of reactions at the injection site (diseases: skin malignancies, allergic and eczematous skin diseases; features: congenital or acquired benign skin lesions (nevus), scars, decorative body modifications (permanent (persistent) drawing, tattoos) applied drawings).<br>20. Participation in any other clinical study within the past 90 days.<br>21. Blood donation (450 ml or more of blood or plasma) less than 2 months before inclusion in the study.<br>22. History of allergic reactions to animal proteins and a tuberculin allergen.
**Exclusion criteria**	The investigator must inform participants that their participation in the study is entirely voluntary and that they have the right to terminate their participation in the study at any time without giving any reason. Information about voluntary participation in the study will be included in the text of the volunteer's Information Leaflet with the Informed Consent Form.<br>A volunteer can withdraw consent to participate in the study at any time without giving a reason. In this case, the volunteer must immediately contact the investigator and inform about his decision to withdraw from the study.<br>Possible reasons for early termination of participation in the study include:<br>1. The investigator decided that the volunteer should be excluded in the interests of the volunteer.<br>2. Mistaken inclusion of a volunteer in the study.<br>3. The decision of the investigator to exclude the volunteer from the study due to a significant deviation from the protocol.<br>4. Volunteers with a questionable reaction to Diaskintest administration of and/or with questionable results of the QuantiFERON TB Gold ELISA test (before the first vaccine administration).<br>5. Adverse event which does not allow the vaccine administration.<br>6. Prescribing therapy from the section "Prohibited concomitant treatment", or restricting the protocol procedures.<br>7. Acute diseases or conditions which, in the opinion of the investigator, require the volunteer to be excluded from the study.<br>8. Refusal of the volunteer from further participation in the study or lack of discipline.<br>9. Failure of the volunteer to attend the visit and loss of communication with him.

Table S2. Demographic characteristics of enrolled participants who received at least one dose of the study drug and had at least one immunogenicity evaluation.

Parameter, statistics	GamTBvac (n = 134)		Placebo (n = 45)
**Age (years)**			
Mean (SD)	28.9 (9.0)		29.6 (8.3)
Median	25.3		26.4
Q1; Q3	21.4; 34.9		22.4; 36.1
min; max	18.0; 49.8		18.2; 47.6
p-value		**0.411**	
**Gender**			
Male	55 (41.0%)		22 (48.9%)
Female	79 (59.0%)		23 (51.1%)
p-value (Fisher)		**0.388**	
**Ethnicity**			
Caucasian	134 (100.0%)		45 (100.0%)
Other	0 (0.0%)		0 (0.0%)
**BMI (kg/m2)**			
Mean (SD)	23.5 (3.0)		23.4 (2.8)
Median	23.5		23.3
Q1; Q3	21.0; 25.4		21.1; 25.6
min; max	18.1; 29.8		18.3; 29.7
p-value		**0.869**	

Table S3. Total adverse events, safety evaluation set, n = 180. X = Number of volunteers with at least one AE; % = Percentage of volunteers with at least one AE in the group; Y = Total number of events.

MEDDRA System Organ Class term<br>MEDDRA Preferred term	GamTBvac (n=135)		Placebo (n=45)	
	X (%)	Y	X (%)	Y
**Total number of volunteers with AEs**	**122 (90.4%)**	**426**	**24 (53.3%)**	**74**
**Blood and lymphatic system disorders**	9 (6.7%)	17	1 (2.2%)	1
Eosinophilia	2 (1.5%)	2	1 (2.2%)	1
Leukocytosis	2 (1.5%)	2	0 (0.0%)	0
Leukopenia	1 (0.7%)	1	0 (0.0%)	0
Lymphocytosis	3 (2.2%)	3	0 (0.0%)	0
Monocytosis	1 (0.7%)	1	0 (0.0%)	0
Neutropenia	2 (1.5%)	2	0 (0.0%)	0
Neutrophilia	2 (1.5%)	2	0 (0.0%)	0
Reticulocytosis	2 (1.5%)	2	0 (0.0%)	0
Thrombocytopenia	2 (1.5%)	2	0 (0.0%)	0
**Gastrointestinal disorders**	**2 (1.5%)**	**3**	**0 (0.0%)**	**0**
Dental caries	1 (0.7%)	1	0 (0.0%)	0
Nausea	1 (0.7%)	2	0 (0.0%)	0
**General disorders and administration site conditions**	**100 (74.1%)**	**215**	**4 (8.9%)**	**5**
Asthenia	1 (0.7%)	1	0 (0.0%)	0
Hyperthermia	1 (0.7%)	1	0 (0.0%)	0

Injection site erythema	17 (12.6%)	20	1 (2.2%)	1
Injection site hematoma	4 (3.0%)	6	0 (0.0%)	0
Injection site induration	17 (12.6%)	1	1 (2.2%)	1
Injection site pain	29 (21.5%)	38	1 (2.2%)	1
Injection site reaction	5 (3.7%)	5	0 (0.0%)	0
Injection site swelling	7 (5.2%)	9	0 (0.0%)	0
Malaise	3 (2.2%)	3	0 (0.0%)	0
Tissue infiltration	1 (0.7%)	1	0 (0.0%)	0
Vaccination site hematoma	2 (1.5%)	2	0 (0.0%)	0
Vaccination site reaction	64 (47.4%)	108	2 (4.4%)	2
**Hepatobiliary disorders**	**0 (0.0%)**	**0**	**1 (2.2%)**	**1**
Hyperbilirubinaemia	0 (0.0%)	0	1 (2.2%)	1
**Infections and infestations**	**2 (1.5%)**	**2**	**2 (4.4%)**	**2**
Herpes virus infection	1 (0.7%)	1	0 (0.0%)	0
Nasopharyngitis	0 (0.0%)	0	1 (2.2%)	1
Respiratory tract infection viral	1 (0.7%)	1	0 (0.0%)	0
Rhinitis	0 (0.0%)	0	1 (2.2%)	1
**Injury, poisoning and procedural complications**	**3 (2.2%)**	**3**	**0 (0.0%)**	**0**
Vaccination complication	3 (2.2%)	3	0 (0.0%)	0
**Investigations**	**72 (53.3%)**	**170**	**22 (48.9%)**	**61**
Alanine aminotransferase increased	7 (5.2%)	8	1 (2.2%)	2
Aspartate aminotransferase increased	12 (8.9%)	13	3 (6.7%)	4
Basophil count increased	1 (0.7%)	1	1 (2.2%)	1
Bilirubin conjugated increased	15 (11.1%)	6	7 (15.6%)	9
Blood bilirubin increased	14 (10.4%)	4	6 (13.3%)	8
Blood creatinine increased	0 (0.0%)	0	1 (2.2%)	1
Blood glucose decreased	1 (0.7%)	1	0 (0.0%)	0
Blood potassium increased	1 (0.7%)	1	0 (0.0%)	0
Blood urea decreased	1 (0.7%)	1	1 (2.2%)	1
Blood urea increased	1 (0.7%)	1	0 (0.0%)	0
Body temperature increased	4 (3.0%)	4	1 (2.2%)	2
C-reactive protein increased	33 (24.4%)	44	7 (15.6%)	12
Eosinophil count increased	4 (3.0%)	4	4 (8.9%)	5
Eosinophil percentage increased	0 (0.0%)	0	1 (2.2%)	1
Hematocrit decreased	1 (0.7%)	1	0 (0.0%)	0
Hemoglobin decreased	7 (5.2%)	7	1 (2.2%)	2
Lymphocyte count decreased	4 (3.0%)	4	2 (4.4%)	2
Lymphocyte percentage decreased	1 (0.7%)	1	0 (0.0%)	0
Monocyte count increased	1 (0.7%)	2	1 (2.2%)	1
Neutrophil count decreased	2 (1.5%)	3	0 (0.0%)	0
Neutrophil count increased	10 (7.4%)	11	3 (6.7%)	4
Neutrophil percentage decreased	1 (0.7%)	1	0 (0.0%)	0
pH urine increased	1 (0.7%)	1	0 (0.0%)	0
Platelet count decreased	1 (0.7%)	1	0 (0.0%)	0
Protein total increased	0 (0.0%)	0	2 (4.4%)	2
Red blood cell count decreased	1 (0.7%)	1	0 (0.0%)	0

Red blood cell sedimentation rate increased	1 (0.7%)	1	1 (2.2%)	1
Urinary nitrogen increased	1 (0.7%)	1	0 (0.0%)	0
White blood cell count decreased	6 (4.4%)	7	2 (4.4%)	2
White blood cell count increased	0 (0.0%)	0	1 (2.2%)	1
**Metabolism and nutrition disorders**	**3 (2.2%)**	**3**	**0 (0.0%)**	**0**
Hyperkalemia	3 (2.2%)	3	0 (0.0%)	0
**Musculoskeletal and connective tissue disorders**	**4 (3.0%)**	**6**	**2 (4.4%)**	**2**
Myalgia	3 (2.2%)	3	2 (4.4%)	2
Myosclerosis	3 (2.2%)	3	0 (0.0%)	0
**Nervous system disorders**	**3 (2.2%)**	**3**	**0 (0.0%)**	**0**
Headache	3 (2.2%)	3	0 (0.0%)	0
**Respiratory, thoracic, and mediastinal disorders**	**3 (2.2%)**	**3**	**2 (4.4%)**	**2**
Cough	1 (0.7%)	1	0 (0.0%)	0
Respiratory disorder	2 (1.5%)	2	2 (4.4%)	2
**Vascular disorders**	**1 (0.7%)**	**1**	**0 (0.0%)**	**0**
Hypotension	1 (0.7%)	1	0 (0.0%)	0

Table S4. Percentages of volunteers with positive specific IgG responses to immunization with GamTBvac. Results were counted as positive if the value was higher than baseline (day 0 mean + 2SD).

**Antigen**	**units**	**days post-vaccination**						
		**21**	**57**	**64**	**78**	**87**	**120**	**150**
Ag85a		9	12	21	50	45	33	26
DBD		19	15	43	81	81	76	64
fusion DBD-Ag85a	%	24	18	55	94	93	85	74
ESAT6		1	2	10	21	18	9	5
CFP10		23	14	53	97	97	97	95
DBD		19	15	43	81	81	76	64
fusion DBD-ESAT6-CFP10		34	26	76	98	98	98	97

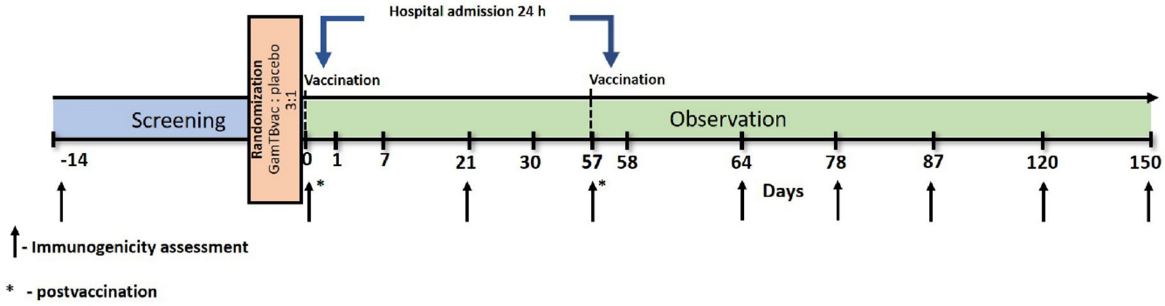

Figure S1. Study design and procedures.

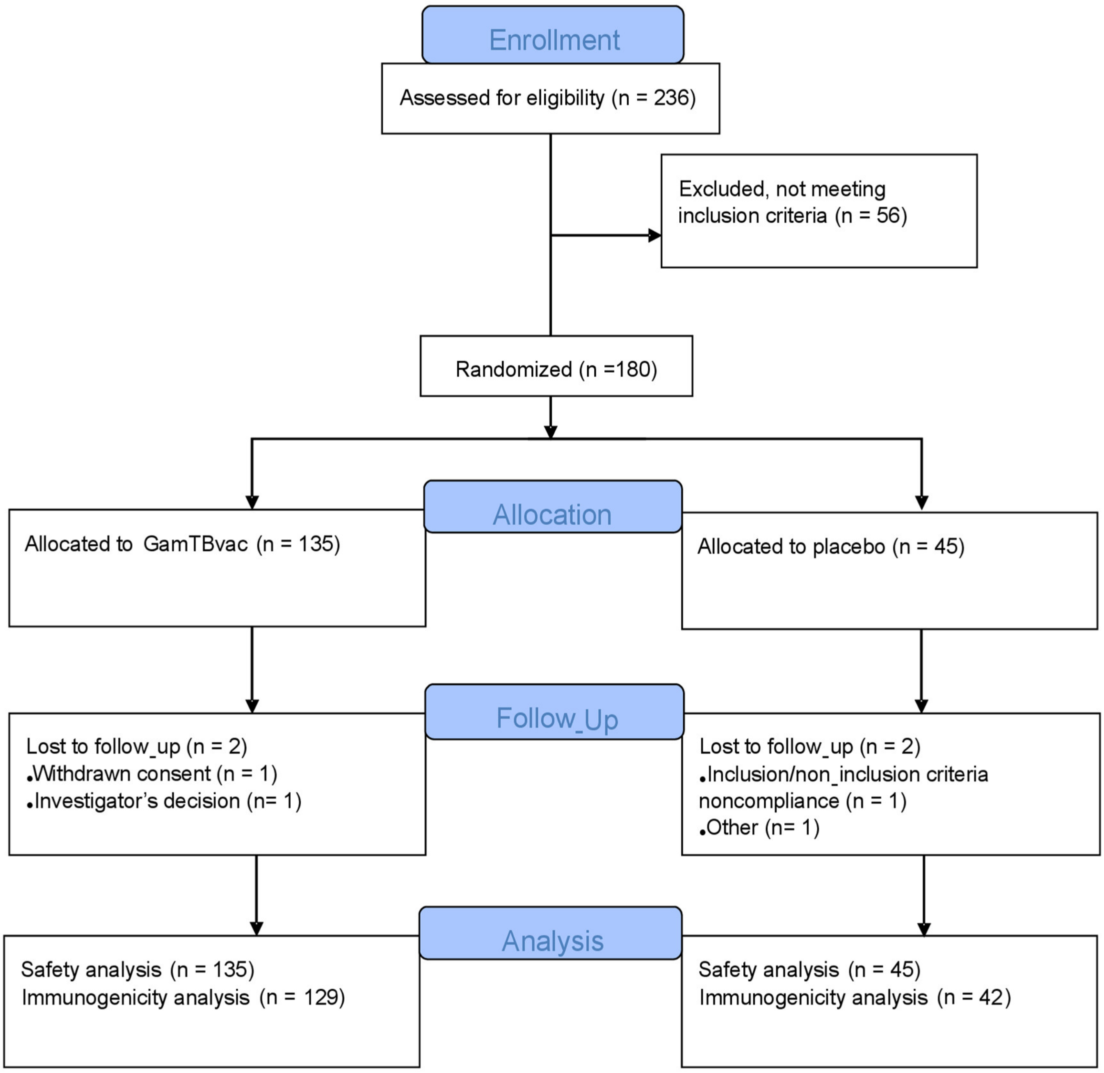

Figure S2. CONSORT diagram of study flow.

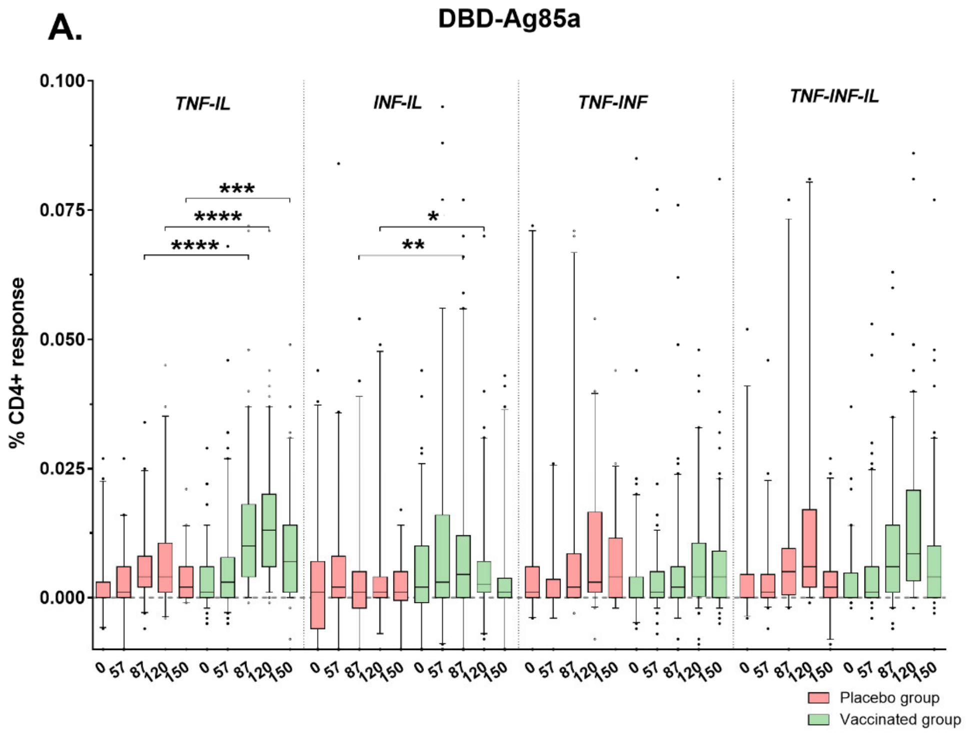

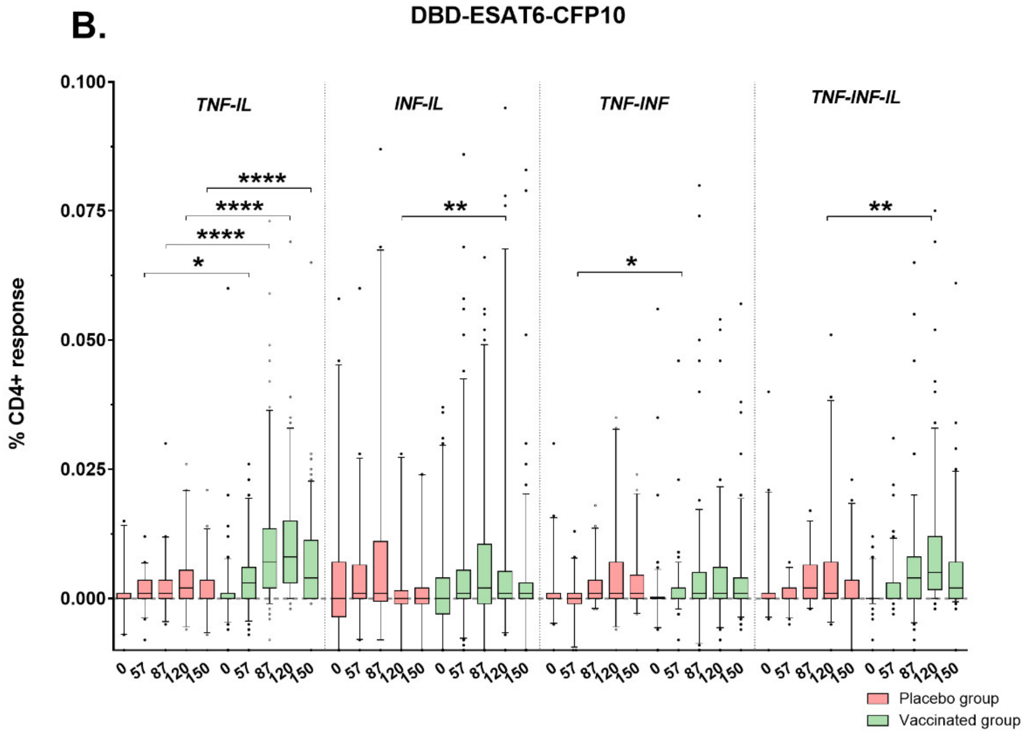

Figure S3. CD4⁺ T-cell responses after stimulation with both fusions of the GamTBvac vaccine in volunteers and the placebo group at various study days 0, 57, 87, 120, and 150. A. Stimulation of samples with DBD-Ag85a; B. Stimulation of samples with DBD-ESAT6-CFP10. Medians and interquartile ranges are shown. **p < 0.05, ** p < 0.01, *** p < 0.001, **** p < 0.0001.*
